# Are videogame training gains specific or general?

**DOI:** 10.3389/fnsys.2014.00054

**Published:** 2014-04-08

**Authors:** Adam C. Oei, Michael D. Patterson

**Affiliations:** Division of Psychology, School of Humanities and Social Sciences, Nanyang Technological UniversitySingapore, Singapore

**Keywords:** video games, transfer (psychology), cognition, perception, learning

## Abstract

Many recent studies using healthy adults document enhancements in perception and cognition from playing commercial action videogames (AVGs). Playing action games (e.g., *Call of Duty*, *Medal of Honor*) is associated with improved bottom-up lower-level information processing skills like visual-perceptual and attentional processes. One proposal states a general improvement in the ability to interpret and gather statistical information to predict future actions which then leads to better performance across different perceptual/attentional tasks. Another proposal claims all the tasks are separately trained in the AVGs because the AVGs and laboratory tasks contain similar demands. We review studies of action and non-AVGs to show support for the latter proposal. To explain transfer in AVGs, we argue that the perceptual and attention tasks share common demands with the trained videogames (e.g., multiple object tracking (MOT), rapid attentional switches, and peripheral vision). In non-AVGs, several studies also demonstrate specific, limited transfer. One instance of specific transfer is the specific enhancement to mental rotation after training in games with a spatial emphasis (e.g., *Tetris*). In contrast, the evidence for transfer is equivocal where the game and task do not share common demands (e.g., executive functioning). Thus, the “common demands” hypothesis of transfer not only characterizes transfer effects in AVGs, but also non-action games. Furthermore, such a theory provides specific predictions, which can help in the selection of games to train human cognition as well as in the design of videogames purposed for human cognitive and perceptual enhancement. Finally this hypothesis is consistent with the cognitive training literature where most post-training gains are for tasks similar to the training rather than general, non-specific improvements.

## Introduction

Over the last decade, effects of videogame play on human perception and cognition have been intensely studied and debated. Most studies have examined effects from action videogame (AVG) play. With a few exceptions (e.g., Boot et al., 2008; Irons et al., 2011), results from independent laboratories have shown experienced AVG players outperforming non-players in a variety of cognitive and perceptual tasks (e.g., Green and Bavelier, 2003; Colzato et al., 2010; Vallett et al., 2013).

What type of games can be considered an AVG? While the complexity and cross-fertilization across videogames makes pigeonholing each game into a distinct category difficult and somewhat arbitrary, AVGs contain many characteristics that make them unique. These include unpredictability, fast speed in presentation and response requirements, high perceptual load, the selection between multiple action plans and an emphasis on peripheral processing (Green et al., 2010a; Hubert-Wallander et al., 2011). Most of the games used in AVG studies have been first-person shooters (FPS) like *Call of Duty*, *Counterstrike*, *Unreal Tournament* and *Medal of Honor* (see also Latham et al., 2013 for more detailed descriptions of different AVGs). Although games of other genres like role-playing (e.g., *Final Fantasy*), puzzle (e.g., *Tetris*) may have one or two features in common with AVGs (e.g., speeded responses), they rarely, if ever, present these all the aforementioned demands in combination. Note that exactly what part of the AVG that leads to transfer is not yet clearly understood, and whether all or only some of the components are necessary for the transfer effects that have been observed.

Although cross-sectional comparisons suggest playing videogames leads to cognitive enhancements, they actually have little bearing on causality (Boot et al., 2011; Kristjánsson, 2013). Primary problems include issues of directionality (i.e., it is unclear whether people develop superior cognitive skills because of gaming or whether people with superior skills become gamers) and expectancy effects (people recruited for their gaming expertise are more motivated and expect to perform better) (Boot et al., 2011; Kristjánsson, 2013).

In contrast to cross-sectional studies, longitudinal-type training studies that show improved cognitive and perceptual abilities following a short bout of videogame training involving novice videogame players make stronger inferences for causality (e.g., Green and Bavelier, 2003; Wu and Spence, 2013). These games used for training are so intriguing because they were not specifically designed with the goal of training human cognition and perception (e.g., Klingberg et al., 2005; Jaeggi et al., 2011; Anguera et al., 2013). Rather, they are commercially available games designed for entertainment. Hence, learning as a result of playing these games is incidental rather than intentional.

## Purpose and scope of the review

While there are many studies documenting the effects of videogame play on cognitive and perceptual skills, the mechanism of transfer is not well understood. One proposal to explain the wide range of AVG-related transfer across multiple perceptual, attentional and executive measures is that the transfer is due to a general improvement in probabilistic inference. In other words, AVG trainees become better able to use evidence from repeated presentations of a task to guide their decision-making and allocation of cognitive resources (Green et al., 2010b; Bavelier et al., 2012b). Hence, AVG experience may enhance a general capacity to control top-down attention and learning of a new task, which in turn translates to improvement across many different tasks. This process is termed “learning to learn” (Bavelier et al., 2012b). Although intriguing, this suggestion is not without problems. First, it is unclear whether this transfer to a general statistical learning ability applies only to AVG or whether it can also be used to explain transfer effects from other videogames. If only applicable to AVG-based learning, it remains unclear what is special about AVG or the exact properties that would be required in an AVG to cause transfer. Second, this hypothesis is too general such that it is not clear which tasks AVG training can and cannot transfer to. Third and most importantly, although it has been demonstrated that AVG trainees do indeed improve probabilistic inference in a visual perceptual task (Green et al., 2010b), empirical evidence is currently lacking to show that this can also account for transfer to the other tasks seen in the AVG literature.

In contrast to the view of Bavelier et al. (2012b), we argue that transfer is task-specific and limited to perceptual and cognitive skills common to both the trained videogame and laboratory transfer task. The roots of this proposal go back to the theory of identical elements (Thorndike and Woodworth, 1901). Therefore, repeated playing of a videogame allows the player to hone the shared specific demands. We argue that the tasks used to test transfer have similar demands to what is trained by AVG. To demonstrate this, we will review each task that has been improved by AVG playing and explain how demands within the AVG are similar to the task itself. The hypothesis of common demands is also consistent with evidence showing that transfer from training is more likely if training and transfer task recruited common neural regions (Dahlin et al., 2008).

The review covers both cross-sectional comparisons between experienced videogame players as well as longitudinal-type training studies. Although videogame training has been studied across the lifespan from young children (Subrahmanyam and Greenfield, 1994; Yuji, 1996) to old adults (Basak et al., 2008), Due to limited space, we limit this review to young adults, which make up the majority of the samples used in the videogame literature.

The reader should note that because of the interest generated by the groundbreaking work that emerged from the Bavelier lab (Green and Bavelier, 2003), the majority of investigations over the last decade have been focused mainly on AVGs. In contrast, non-AVGs are rarely studied. Rather, non-AVG players and non-AVG training groups are often used as control groups. Hence, inevitably a large portion of the review will document cognitive and perceptual enhancements via AVG play. Nevertheless, where available, we review evidence for transfer effects arising from non-AVGs as these studies also provide evidence to support our common demands hypothesis. We review the evidence for videogame-related transfer starting from lower level perceptual skills to higher-order cognitive control.

## Evidence for videogame-related transfer

Enhancements in many visual-perceptual skills have been demonstrated empirically in many AVG-training studies and those that compared non-AVG and habitual AVG players. The different demands described above in AVGs allow predictions on what abilities are trained and the plausible transfer effects using the common demands hypothesis. Consider what is expected in a typical AVG. In these games, players often are required to detect and respond to enemies quickly as well as keeping track of them as they move around the screen. These demands are coupled with the need to attend to several items simultaneously in both central and peripheral vision. These are similar to the demands in multiple object tracking (MOT) and Useful Field of View (UFOV) tasks. Moreover, as enemies appear rapidly one after another or simultaneously, there is great emphasis on the ability to rapidly switch attention from one target to another. This is similar to an attentional blink task. In addition, players have to resist being distracted by task irrelevant stimuli no matter how salient these distractors are. This may lead to improved performance in tasks that require suppressing distractors. Failure to successfully perform any of the above may result in failure in the game mission. Because of these special properties, one can imagine that hours spent on playing action AVG play can serve to exercise many of these perceptual and attentional skills that underpin successful gameplay.

### Contrast sensitivity

An important aspect of visual perception that is enhanced by AVG playing is the ability to detect subtle contrast differences. Specifically, Li et al. (2009) showed that habitual AVG players outperformed non-videogame players in the ability to detect a low contrast Gabor patch. Additionally, videogame novices also showed enhancements in this skill following 50 h training in a fast-paced FPS relative to playing a control non-action game (*The Sims*).

At first glance, it does not appear obvious that an AVG experience demands detecting contrast differences between objects. However, note that in many action games, a strong emphasis is on distinguishing targets from non-targets to allow rapid responding. The emphasis on rapid responding thus places a demand on distinguishing a target from a non-target based on even the subtlest differences (e.g., visual characteristics such as color or contrast).

### Peripheral vision

AVGs make good candidates for training peripheral vision because of their heavy emphasis on detecting targets across different central and peripheral areas. For example, in many shooter games, enemies often appear at far areas of the periphery and players must spot and dispatch them early to advance in the game. It is therefore likely that hours spent on AVG play would serve to enhance sensitivity to targets in the periphery.

One measure of peripheral vision is the UFOV, which is the total area of the visual field where useful information is captured at a glance without eye or head movements (Sanders, 1970; Ball et al., 1988). Comparisons of UFOV between regular AVG players and non-players have shown that the former exhibited superior ability to detect targets at peripheral areas of vision (10^°^, 20^°^ and 30^°^ eccentricity) (Green and Bavelier, 2003). Target detection at these eccentricities was also enhanced following AVG training for as short as 10 h relative to *Tetris* (Green and Bavelier, 2003). This enhanced target detection in regular AVG players and non-players trained to play an AVG was also found in more demanding UFOV tasks that included more distractors and a secondary task (Green and Bavelier, 2006a).

As the UFOV represents only an effective or functional field of view relevant to a particular visual task, videogame effects on peripheral vision have also been studied using clinical measures of central and peripheral visual fields. The results in Green and Bavelier (2003, 2006a), where videogame advantage extended to 30^°^ eccentricity from fixation may have represented only the outer edges of central vision (Buckley et al., 2010). Hence, Buckley et al. (2010) tested regular AVG players and non-players on the Goldman Kinetic perimetry, a standard clinical measure of peripheral vision. Their results replicated Green and Bavelier (2003, 2006a) by showing enhanced central visual fields (30^°^ eccentricity from fixation). Crucially, the AVG players also had, on average, a larger peripheral visual field (60^°^ eccentricity from fixation). These results taken together provide strong evidence of AVG-related enhancements to peripheral vision, which may have ecological significance especially given that gender differences in UFOV can be reduced with AVG training (Feng et al., 2007).

Although the aforementioned studies show an AVG-related enhancement in UFOV, other studies have failed to replicate these results. Specifically, Boot et al. (2008) and Murphy and Spencer (2009) found equivalent UFOV performance in AVG and non-AVG players. Furthermore, no differences were found in UFOV improvement beyond test-retest effects in AVG trainees after 21.5 h of training relative to those trained in a strategy game and *Tetris* (Boot et al., 2008). The reason for the discrepancies in findings is unclear, but in cross-sectional comparisons, differences in selecting samples may have resulted in the null findings. For example, in Boot et al. participants in the AVG group did not exclusively play that genre but also reported playing other genres as well. As for the training study, Strobach et al. (2012) speculated that the large number of transfer tasks in their training study might have undermined any transfer effects due to test fatigue. Also, it is important to note that the transfer tasks in Boot et al. were administered three times at pre, mid and post-training. Presumably, multiple testing induced practice effects in the AVG and control groups that masked transfer effects.

### Divided attention

Given the nature of many fast-paced AVG, the ability to divide attention to several items confers a great advantage when playing these games. Hence, according to the common demands hypothesis playing fast-paced AVG should potentially enhance performance in tasks that require allocation of attention towards several items. Current evidence generally supports this claim (but see Boot et al., 2008).

#### Posner cueing

In one of the first studies that demonstrated spatial attentional advantages in videogame players, Greenfield et al. (1994) showed that expert players of the game *Robot Battle*, were superior in attending to more than one space relative to game novices using the Posner cueing task (Posner et al., 1980). Briefly, in the Posner cueing task, a cue was given to indicate the probable location of a target. There were three probabilities of the target appearing where the cue indicated—80% (high probability), 50% (neutral) or 20% (low). The speed of target detection was fastest in the high probability condition, and slowest in the low probability condition (Posner et al., 1980). In terms of overall response times, expert videogame players were faster in target detection in the high and low probability conditions compared to non-gamers. Furthermore, the videogame experts did not show an increased response time in the low probability relative to the neutral condition (Greenfield et al., 1994).

Demonstrating a causal effect, those trained to play a videogame, *Robotron*, for 5 h, where a player fended off robot attacks from many directions, showed greater improvement at the low probability condition whereas non-players showed no improvement. These results thus provided early and preliminary evidence of AVG-related advantage in attending to multiple locations in space, a demand common to many fast-paced AVG.

#### Flanker effects

Corroborating evidence for AVG-related superior attentional capacity to attend to more items in parallel has also been shown using different attentional tasks. In their groundbreaking work using a modified flanker task (Lavie and Cox, 1997), Green and Bavelier (2003) claimed that experienced AVG players had leftover attentional resources to attend to distractors when performing a demanding task, whereas non-gamers did not attend to them and thus were not distracted. This suggests that AVG players had greater attentional capacities to attend to multiple items in parallel. In contrast, Irons et al. (2011) failed to replicate these results. However, note that both studies were cross-sectional, not training studies. Hence, it is unclear whether the AVG advantage is causal. The discrepancy in findings may simply reflect sample differences. A training study is therefore needed to resolve this issue.

#### Multiple object tracking

Green and Bavelier (2006b) found that experienced AVG players were able to track on average two items more than non-gamers in an MOT task. In addition, following 30 h of training, non-players trained in an action game, *Unreal Tournament* improved accuracy rate when the number of objects to-be-tracked increased beyond four items. In contrast, accuracy rate for the control group trained in *Tetris* remained unchanged regardless of the number of targets shown. Similar enhancements were found after 20 h of training using a different measure of MOT (Oei and Patterson, 2013). Corroborating these results, Boot et al. (2008) showed that AVG players were able to track on average, at higher speeds than non-gamers.

The transfer to MOT appears confined to fast-paced FPS. In contrast, training using slower-paced AVGs or other types of AVG such as fast-paced sports game showed no such improvement (Cohen et al., 2008). Like MOT tasks, FPS have multiple fast moving objects that require simultaneous tracking. In contrast, games with multiple items on screen without requiring attention to-be-allocated to all items simultaneously (such as match-3 games) may not result in transfer after training (Oei and Patterson, 2013). However, this hypothesis remains tentative as Boot et al. (2008) failed to find evidence for enhanced MOT tracking speed in participants trained in a fast-paced FPS for 21.5 h compared to non-AVG trained participants.

#### Enumeration

Evidence for the enhanced ability of AVG players to attend to multiple items has also been corroborated using an enumeration task. Green and Bavelier (2006b) showed that experienced action gamers were more accurate in estimating the number of items displayed than non-action gamers. In addition, experienced action gamers were able to estimate about two items more than non-action gamers. This enhanced ability was also found after 10 h training using an FPS AVG relative to controls (*Tetris* training).

### Visual search

FPS games are highly similar to a visual search paradigm because players must search for targets amidst distractors, such as an enemy in hiding (Wu and Spence, 2013). Indeed, converging evidence shows search advantages in habitual AVG players. First, AVG players searched faster and more efficiently overall (Hubert-Wallander et al., 2011) without sacrificing accuracy (Castel et al., 2005). Additionally, AVG players searched more accurately and faster in demanding conjunction conditions (Wu and Spence, 2013). Finally, AVG players were able to search more accurately when distracting objects were in close proximity to the target (Green and Bavelier, 2007), a condition known as crowding (Toet and Levi, 1992; Intriligator and Cavanagh, 2001).

Several longitudinal studies have corroborated the cross-sectional results. These studies used both FPS and other types of AVG training compared with non-AVG training. In a 30-h training study, Green and Bavelier (2007) found that players trained in an AVG (*Unreal Tournament*) were able to detect targets at reduced target-distractor separations compared to the non-AVG group (*Tetris*) at 0^°^, 10^°^ and 25^°^ eccentricity. Furthermore, following 10 h of training using an FPS, and a racing AVG compared to a control group (3D puzzle game, *Ballance*), Wu and Spence (2013) demonstrated greater accuracy and faster search time for both types of AVG players compared to the control game in a dual search task that involved searches in central and peripheral vision. Interestingly, the results for the FPS and racing game were equivalent. Although most racing games do not include visual search, Wu and Spence (2013) argued that for this particular racing game, the player was expected to also locate and identify several targets. Therefore, these results match the common demands hypothesis.

Demonstrating that not all AVG are alike, we failed to find an AVG-related enhancement in visual search following 20 h of FPS training (Oei and Patterson, 2013). The AVG game used in Oei and Patterson did not have search demands because enemies tended to pop out and engage the player rather than making the player search them out.

Importantly, Oei and Patterson (2013) showed that transfer is not dependent on the training game being an AVG. Instead, visual search time was significantly decreased following training in a hidden object game and a match-3 game (*Bejeweled*) that required searching a display for matching shapes. Therefore, the data in Oei and Patterson (2013) and Wu and Spence (2013) support the common demands hypothesis, such that training in games that included frequent search improved visual search performance. On the other hand, the failure to find evidence for AVG related transfer is inconsistent with a general transfer mechanism, which would lead to improvements across multiple tasks, including visual search.

### Change detection

A fundamental requirement in any fast-paced AVG is the need to respond quickly to a sudden onset stimulus. This could be a visual anomaly such as an enemy that appears when the player is preoccupied by something else in the visual field. Given this requirement for successful gameplay, it is plausible that expert AVG players will exhibit superior ability to detect visual anomalies when they are focused on other features in their visual field.

Current evidence for enhanced change detection in AVG players remains mixed. On one hand, Murphy and Spencer (2009) showed that AVG and non-AVG players are equally as likely to miss a cross moving across their visual field while performing a counting task. On the other hand, Vallett et al. (2013), using a popular inattentional blindness task (Simons and Chabris, 1999), showed that AVG players have a significantly greater likelihood of detecting a visual anomaly (man in a gorilla suit) while performing a counting task than non-gamers. One possibility for the inconsistent finding is that the original task in Simons and Chabris presented a more salient visual anomaly than that in Murphy and Spencer. This is not unlike an AVG where a sudden-onset visual stimulus is likely to be a salient one that demands a response (e.g., enemy).

### Attentional blink

Attentional blink refers to a bottleneck in information processing whereby a second target (T2) presented close in time (200–500 ms) to an accurately detected first target (T1), fails to be detected (Raymond and Shapiro, 1992; Shapiro et al., 1994, 1997). Green and Bavelier (2003) reported that AVG players were less affected by attentional blink than non-gamers. Furthermore, non-gamers trained in an AVG for 10 h improved T2 detection during the intervals susceptible to the attentional blink effect (Green and Bavelier, 2003). This training-related enhancement specifically on a fast-paced FPS has generally been replicated following 12–20 h of training (Cohen et al., 2008; Oei and Patterson, 2013). Crucially, the enhancement is seen only following a fast-paced FPS, but not to other slower paced FPS, third-person shooters and sports games (Cohen et al., 2008). Compared to other AVG, fast-paced FPS requires fast responses to rapidly presented successive targets which leads to these specific improvements (Cohen et al., 2008). This is consistent with the common demands hypothesis.

In contrast, Boot et al. (2008) and Murphy and Spencer (2009) failed to find AVG-related advantages in attentional blink performance. Importantly, no attentional blink enhancement was seen beyond test-retest effects in AVG trainees after 20 h of training (Boot et al., 2008). However, it is again important to place the findings in context of the criticisms of the study mentioned above.

### Spatial cognition

Spatial cognition involves multiple components and broadly speaking refers to the skill in representing, transforming, generating and recalling symbolic, nonlinguistic information (Linn and Petersen, 1985). AVG, especially those with a first-person perspective, should be good training tools for some spatial skills due to navigation and rotation demands in 3D space (Spence and Feng, 2010; Sanchez, 2012). However, training may not transfer to other components if the relevant spatial cognitive demands are not present in the game (Okagaki and Frensch, 1994). For example, enhanced 3D mental rotation was found only following FPS training but not using games without demands to navigate in 3D space (Feng et al., 2007; Sanchez, 2012). Furthermore, the enhancement to spatial ability was specific only to mental rotation of 3D shapes but not to spatial visualization in a paper-folding task where no rotation was necessary (Sanchez, 2012).

Transfer is also seen using non-AVG training. Specifically, faster and more accurate mental rotation has been found in experienced and trained *Tetris* players (Okagaki and Frensch, 1994; Sims and Mayer, 2002; Boot et al., 2008). Unlike AVGs, *Tetris* does not involve fast responding except for the highest levels. Furthermore, new objects in *Tetris* always enter from one location (vertically from the top) and hence there is no requirement to track multiple moving objects in the periphery. There is also no requirement to construct a representation of a complex 3D environment in order to navigate. Finally, virtually no distractors appear in *Tetris*. Rather, participants must stack falling shapes efficiently using mental rotation and planning. These demands lead to different, specific effects compared to those commonly found from playing action games, indicating that *Tetris* should be classified a non-action game.

Three months of *Tetris* training was associated with decreased activation in right frontal (BA 32, 8, 9, 6, 46) as well as parietal (BA 40) areas (Haier et al., 2009). These areas have been previously been shown to be highly activated in mental rotation tasks (Cohen et al., 1996). This reduction in brain activation may suggest enhanced neural efficiency to perform mental rotation (cf. Haier et al., 1992).

Transfer effects from *Tetris* training were highly specific to mental rotation measures (Boot et al., 2008). Notably, the shapes used in the mental rotation task resembled shapes that appeared in *Tetris* (Boot et al., 2008). Conversely, no advantage was seen in skilled or trained players in mental rotation tests that did not involve *Tetris*-like shapes (Sims and Mayer, 2002; Boot et al., 2008). Furthermore, although 12 h of *Tetris* training did not result in transfer to spatial ability tests in general, examinations of the mental rotation strategies showed that *Tetris* trainees were more likely to use a Tetris-like mental rotation (clockwise rotation up to 225^°^) for *Tetris* shapes (Sims and Mayer, 2002).

These specific improvements thus add converging evidence to the proposal of a transfer being more likely if the game and transfer task share common demands. In contrast, a more general transfer mechanism would predict a general spatial ability or overall mental rotation enhancement. Moreover, the general learning proposal was originally proposed to explain AVG-related improvements only. Thus, the common demands theory allows explanation of more types of video-game related cognitive changes.

### Executive functions

#### Task switching

Both alternate-runs and random task switching paradigms have been employed to study the effects of videogame playing on task switching. In the former, a task-switch takes place after a fixed number of trials allowing preparation so that switching is less demanding and typically yields smaller switch costs (Monsell, 2003). In contrast, task-switches are random and unpredictable in the latter. The inability to prepare for a switch leads to greater conflicts, which in turn results in the switch cost being larger (Rogers and Monsell, 1995; Monsell, 2003).

Smaller switch costs in reaction time (RT) and accuracy have been demonstrated in regular AVG players compared to non-players in both alternate-runs and more demanding random task switches (Andrews and Murphy, 2006; Boot et al., 2008; Colzato et al., 2010; Cain et al., 2012; Green et al., 2012; Strobach et al., 2012). Greater switch cost reductions in alternate-runs task switching was found following 15 (Strobach et al., 2012) and 50 h (Green et al., 2012) of AVG training compared to controls that played non-AVGs (e.g., *Tetris* and *The Sims*).

Although task switching superiority in experienced AVG players is consistently reported, results of training studies for transfer to task switching remain equivocal. In contrast to Green et al. (2012) and Strobach et al. (2012), Boot et al. (2008) failed to find transfer effects following 21.5 h of AVG training. Different task-switching paradigms used in each study may explain the conflicting results. Specifically, while Green et al. (2012) and Strobach et al. (2012) utilized a predictable alternate-runs switch format, Boot et al. (2008) instead used a random task switching paradigm. Thus, AVG training may only improve the ability to prepare for upcoming switches but not more demanding mental flexibility measured by random task switching paradigms as these are supported by different neural and cognitive mechanisms (see Baddeley et al., 2001; Bryck and Mayr, 2005; Pereg et al., 2013).

Consistent with the argument of the common demands hypothesis, the transfer of predictable task switching may stem from the frequent practice in AVGs to switch between targets and between items during gameplay. Each activity has clear objectives with little conflict between them. Thus disengagement from a previous task to switch focus on an upcoming task is relatively easy. Take for instance switching rapidly between enemies where the switch can be planned or reactive. Either way, the action following a switch is similar. This is like a task-repeat and involves negligible response conflict. Even switching from an enemy to collecting an item (e.g., health replenishments or bonus items) is unlikely to result in any decisional or response conflicts as the actions involved with either task are distinct. These are akin to the predictable task switching condition whereby any switch is predictable and an upcoming switch or task-repeat condition can be planned in advance. In contrast, decisional conflicts like the random task switching condition are rarely, if ever, encountered in such AVGs.

#### Distractor suppression

Several studies indicate that AVG players are less susceptible to attentional capture by task-irrelevant stimuli than non-players. Experienced AVG players and those trained to play an AVG for 20 h were faster in responding to targets in the presence of distractors (Chisholm et al., 2010; Oei and Patterson, 2013). This advantage may stem from improved top-down suppression of attentional capture rather than a faster recovery from capture (Chisholm and Kingstone, 2012). Support for better distractor suppression in AVG players comes from neuroimaging. Relative to non-gamers, action gamers showed increased suppression of steady state visually evoked potential (SSVEP) amplitudes to unattended peripheral stimuli in a target detection task where the goal was to detect targets at central fixation or when cued, at left and right peripheries (Mishra et al., 2011). Additionally, using a visual search task with moving distractors at central or peripheral vision, action gamers showed reduced blood-oxygenated level dependent (BOLD) response in visual motion-sensitive regions (Medial Temporal/Medial Superior Temporal areas) to moving distractors compared to non-gamers (Bavelier et al., 2012a). Moreover, 10 h of AVG training has been shown to increase P2 and P3 waves at occipital and occipito-parietal sites (Wu et al., 2012) when performing an attention visual field task where one is required indicate the direction of a target amidst distractors. Increases in P2 and P3 amplitudes may reflect adaptations to task demands on attentional control in attentional selection as well as inhibition of processing of task-irrelevant stimuli (Bledowski et al., 2004; Potts et al., 2004; Sawaki and Luck, 2010; Fritzsche et al., 2011). Taken together, these findings suggest that experienced AVG players are better than their non-AVG counterparts at applying top-down control to suppress attention for task-irrelevant distractors. Longitudinal training studies further show that the advantages displayed are causal.

## Specific or general transfer? The case for specific transfer gains

Over the last 10 years since the seminal work of Green and Bavelier (2003), the focus of videogame training has been directed towards AVGs. The growing literature suggests that AVGs enhance lower-level information processing skills ranging from visual perception to different aspects of attention (Green and Bavelier, 2003, 2006a,b, 2007; Chisholm et al., 2010). These include expanded peripheral vision (Green and Bavelier, 2003), target discrimination, identification and contrast (Green and Bavelier, 2007; Li et al., 2009), selective attention (Wu et al., 2012) and attentional blink (Cohen et al., 2008; Green and Bavelier, 2003). In contrast, evidence for transfer to some executive functions remains equivocal (e.g., Boot et al., 2008).

Given the differing characteristics of non-action games, one would expect different types of transfer from non-action game training compared to AVG. However, unlike AVGs, not much research has been conducted to determine the range of transfer. Nevertheless, a small number of studies also suggest some benefits from non-AVGs such as *Tetris’* transfer to mental rotation (Okagaki and Frensch, 1994; Sims and Mayer, 2002; Boot et al., 2008). Importantly, like AVG, transfer effects are also quite specific to skills that are common to the trained game and transfer task (Okagaki and Frensch, 1994; Sims and Mayer, 2002; Boot et al., 2008; Oei and Patterson, 2013).

A critical question we set out to answer in this review is whether transfer gains from videogames reflect a general or more specific enhancement. One hypothesis is that a general attentional control mechanism accounts for transfer effects seen in (action) videogames. This theory suggests that (action) videogame play enhances a general learning of task statistical patterns that supports perceptual decision-making and allocation of cognitive resources (Green et al., 2010b; Bavelier et al., 2012b). However, it has not been empirically demonstrated that this mechanism can indeed account for transfer across the wide range of tasks utilized in the videogame literature. Moreover, it is unclear if such a learning mechanism is applicable to non-action games. Furthermore, the data reviewed earlier do not support this hypothesis. Specifically, if a general learning mechanism really underlies AVG-related transfer, enhancements should be seen across multiple tasks, even those that do not share overlapping demands with the trained AVG. Unfortunately, as most current studies only included transfer tasks that share common demands with the trained AVG, it is difficult to assess the validity of this hypothesis. In the few studies that included tasks that do not appear to share common demands with AVG used for training, however, AVG playing did not transfer to all tasks (e.g., Boot et al., 2008; Murphy and Spencer, 2009; Oei and Patterson, 2013).

In contrast to a general transfer mechanism, the main proposal here is that transfer effects are specific to common demands shared between the trained videogame and transfer task. This hypothesis can explain both AVG and non-AVG training effects (Oei and Patterson, 2013).

Converging evidence from neuroimaging also appears to support the current hypothesis. For instance, transfer is more likely if training and transfer tasks recruit overlapping neural regions (Dahlin et al., 2008). Evidence from the working memory training literature also supports specific over general transfer. For instance, working memory training improved performance on working memory measures but not to measures of fluid intelligence (Harrison et al., 2013; Melby-Lervåg and Hulme, 2013; Redick et al., 2013). Hence, we feel that although working memory and videogame play are different activities, they share a common principle in relation to transfer of cognitive skill in that transfer is specific to what is practiced within the training regime. In contrast, little or no transfer can be expected for skills not explicitly practiced.

We do note that although the general transfer mechanism proposed by Bavelier et al. (2012b) contrasts with the common demand specific mechanism proposal, they added a caveat that “changes in knowledge produce benefits only to the extent to which new tasks share structure with AVG. No benefits are expected in tasks that share no such structure” (Bavelier et al., 2012b). No study has yet been reported that systematically manipulates the demands contained in the AVG used for training because the definition of AVG has remained overly general, but there is some preliminary evidence that FPS AVG lead to different results than other types of AVG (Cohen et al., 2008).

## Concluding remarks and future directions

Several gaps and unanswered questions still remain. First, it remains unclear how “closely-matched” the game and transfer task must be to maximize transfer. It is likely that the demands of the training game and transfer task must engage common neural networks (Dahlin et al., 2008). Nevertheless, quantitative metrics of how close a training game and the transfer tasks remains elusive and are worthy of further investigation. A quantifiable metric would be most useful especially in occupational settings in order to guide the selection of a training paradigm that maximizes transfer effects. For instance, in FPS, one could compare games with either progressively increasing speeds in which enemies appear or the number of enemies that appear simultaneously. We would predict that high-speed games would lead to a decrease in attentional blink, and faster RT, but would not have as large of an effect on MOT as the latter manipulation. To further test for the specificity of transfer effects, one could also conduct a study whereby games with different demands (or intensity) from the same genre are compared (see Cohen et al., 2008 for such an example). To determine whether general or specific transfer has occurred, it is important to include a wider variety of transfer tasks, some of which contain similar demands to the videogames and some of which measure more general learning. To test for general transfer, a videogame training regime could also target training visual attention or visual working memory and test whether improvements generalize across to the verbal or auditory modalities using material that was not trained during the games.

A second gap relates to which cognitive abilities can or cannot be improved by training. It will be important in the future to investigate which abilities are more resistant or amenable to be modified with training or whether improvement in one area will lead to worse performance in another (Takeuchi et al., 2011).

A third important issue is the durability of transfer effects. Thus far, many videogame studies have not tested whether the transfer effects remained after the laboratory tests have concluded (see Li et al., 2009; Anguera et al., 2013 for exceptions). Hence, it is unclear whether transfer effects remain after cessation of training. A main goal of a training task is the retention of skills in the long-term after the training has ceased (Schmidt and Bjork, 1992). As with any type of training, the effectiveness of videogame training should also be evaluated with this criterion.

Fourth, more research should examine individual differences in training-related transfer. It has been argued that the capacity for “cognitive modifiability” as a result of training varies from individual to individual (Calero and Navarro, 2007). It is thus unlikely that all individuals trained with a similar videogame improve similarly (see Wu et al., 2012). There are many factors that can influence plasticity and how well one responds to a training regime. Briefly, some examples of individual differences shown to influence training, cognition and transfer include age and baseline cognitive ability including intelligence (Yesavage et al., 1988, 1990; Haier et al., 1992; Verhaeghen et al., 1992; Bissig and Lustig, 2007; Calero and Navarro, 2007), gender (Feng et al., 2007) as well as lifestyle factors such as cardiovascular health and exercise (Gomez-Pinilla, 2008). Thus far, videogame training studies have not examined how these individual differences affect training-related transfer. Hence, investigating training-related transfer from an individual differences perspective is worthwhile as it can be critical for the implementation of a training regime to maximize transfer effects.

Finally, we feel that ultimately a training regime should translate to real-world applications outside of the laboratory. Thus far, the bulk of the studies have focused on laboratory tasks. Just like games have several demands in common with the cognitive tasks, we assume many everyday tasks have common demands that are trained by the videogames. Thus, we assume that playing videogames will lead to improvements in activities in everyday life. However, few studies have shown video game-related advantage in real-world activities. Videogame experience and skill has been shown to correlate with laparoscopic surgery skills (Rosser et al., 2007). Also, a short bout (10-h) of videogame training, albeit not a commercially available one, has been shown to improve flight performance in cadet pilots (Gopher et al., 1994). One potential area of future research may be to investigate whether videogame training in search skills can benefit performance in occupations that demand intense visual search skills (e.g., airport baggage scanners or air traffic controllers). Hence, the potential of videogame training advantages in real-world applications warrants further investigation.

In closing, over the last decade, we have seen considerable literature documenting the potential benefits of videogame training. With the increase in attention and effort dedicated to this area, intense debate, skepticism and scrutiny have also resulted (Boot et al., 2011, 2013; Kristjánsson, 2013). Nevertheless, such intense debate and scrutiny can only be beneficial to researchers as they strive to refine the methodology of videogame training research. Despite the increasing number of works and considerable progress, the field is still in its infancy and considerable advances are yet to be made. There are many advantages with training via a videogame. Training via videogame represents a departure from traditional learning activities in that it is highly arousing and motivating and has the potential to keep the player engaged for longer periods. With progress in computing power and artificial intelligence, there are arguably major leaps that can be made in game immersion and realism. Additionally, with input from psychologists and learning theory in videogame design, we can further tailor videogames for learning purposes. Hence, further investments in time and money to understand, research and improve transfer from videogame training to the work place, classroom and rehabilitation is surely worthwhile.

## Conflict of interest statement

The authors declare that the research was conducted in the absence of any commercial or financial relationships that could be construed as a potential conflict of interest.
